# Hepatic glutamic-oxaloacetic transaminase promotes mitochondrial respiration energized at complex II and alters whole body metabolism

**DOI:** 10.1016/j.jbc.2025.110261

**Published:** 2025-05-21

**Authors:** Brian D. Fink, Ritu Som, Adam J. Rauckhorst, Eric B. Taylor, Liping Yu, William I. Sivitz

**Affiliations:** 1 Department of Internal Medicine/Endocrinology and Metabolism, University of Iowa and The Iowa City Veterans Affairs Medical Center, Iowa City, Iowa, USA; 2 Department of Molecular Physiology and Biophysics, University of Iowa, Iowa City, Iowa, USA; 3 Department of Biochemistry and Molecular Biology, University of Iowa, Iowa City, Iowa, USA; 4 NMR Core Facility, Carver College of Medicine, University of Iowa, Iowa City, Iowa, USA

**Keywords:** mitochondria, respiration, mitochondrial inner membrane potential, oxaloacetate, glutamic-oxaloacetic transaminase-2, liver, succinate dehydrogenase, mitochondrial complex II

## Abstract

The mitochondrial enzyme, glutamic-oxaloacetic transaminase (GOT2), catalyzes the reaction between oxaloacetate and glutamate generating aspartate and alpha-ketoglutarate. Glutamate can also be directly converted to alpha-ketoglutarate by glutamate dehydrogenase. We investigated mitochondrial and systemic effects of an inducible liver-specific mouse GOT2 knockout (KO). We observed no differences in body mass or percent fat mass in KO mice; however, KO mice had lower fasting glucose and liver tissue contained more fat. Respiration by liver mitochondria energized at complex II by succinate + glutamate was decreased in KO compared to WT mice at low inner membrane potential (ΔΨ) as induced by titration with ADP. Metabolite studies by NMR showed that at low *versus* high ΔΨ, GOT2KO mitochondria energized by succinate + glutamate generated more oxaloacetate (a potent inhibitor of succinate dehydrogenase) and less aspartate. Respiration and mitochondrial metabolites energized by pyruvate + malate or palmitoyl-carnitine + malate did not differ between KO and WT mice. Respiration by GOT2KO mitochondria energized by glutamate + malate was decreased at all levels of ΔΨ. Pathway analysis of LC-MS profile data in the liver tissue of KO *versus* WT mice revealed differential enrichment of the malate aspartate shuttle, TCA cycle, aspartate metabolism, glutamate metabolism, and gluconeogenesis. In summary, GOT2KO impaired potential-dependent complex II energized O_2_ flux likely due at least in part to oxaloacetate inhibition of succinate dehydrogenase.

The mitochondrial form of glutamic oxaloacetic transaminase (GOT2) catalyzes the reaction between oxaloacetate (OAA) and glutamate to form aspartate and alpha-ketoglutarate (α-KG). Glutamate can also be directly converted to α-KG by mitochondrial glutamate dehydrogenase (GDH). The GOT2 reaction, by OAA conversion to aspartate, diverts OAA from the classical circular tricarboxylic acid (TCA) pathway. However, α-KG concurrently generated by GOT2 enters the cycle downstream from isocitrate dehydrogenase.

OAA is well-known to be a highly potent inhibitor of succinate dehydrogenase (SDH) (1, 2). In past work on muscle and brown adipose tissue (BAT) mitochondria energized by succinate, we provided evidence that OAA, measured by NMR spectroscopy, accumulated at low inner membrane potential (ΔΨ) and inhibited SDH thereby decreasing respiration. However, using similar methodology, we found that OAA production by liver mitochondria was too low to detect. Of note, OAA is very difficult to measure by mass spectroscopy due to instability.

In recent months, we found a way to modify our methodology for measuring OAA in liver mitochondria allowing accurate detection of this metabolite (3). With this in hand, we hypothesized that deleting hepatic GOT2 would result in OAA accumulation leading to impaired mitochondrial respiration energized at complex II. Although this hypothesis is directed at hepatic function, the same question could be directed at brown fat and muscle where we previously showed the effect of OAA to inhibit SDH. However, we could barely, if at all, detect GOT2 in intrascapular brown fat by immunoblotting. GOT2 is clearly expressed in skeletal muscle and we plan separate studies directed at the effects of GOT2 deletion in muscle.

OAA is critical in liver as it is centrally positioned to affect alternate pathways including gluconeogenesis, TCA flow to citrate, or formation of aspartate, a critical amino acid required for DNA synthesis (4). Although OAA is low in abundance, the direct products of OAA metabolism (citrate, aspartate, α-ketoglutarate, and malate) are far more abundant implying that OAA turnover is a critical determinant of cellular metabolism. Given this importance, we were interested in how GOT2 catalysis of OAA to aspartate might affect liver mitochondrial O_2_ flux and overall metabolite flow. Here we investigated the role of GOT2 using an inducible liver-specific GOT2KO on respiration by hepatic mitochondria in mice energized by different substrates at different levels of ΔΨ and on associated changes in whole-body metabolism.

## Results

### Mouse characteristics

**Figure 1:**
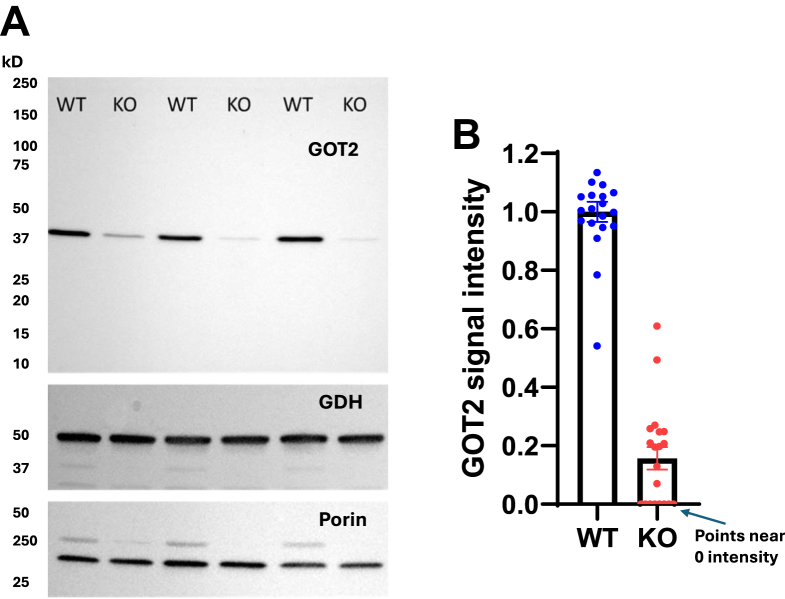
**GOT2 expression in the liver mitochondria of WT and GOT2KO mice.***A*, representative immunoblot depicting GOT2, GDH, and porin expression in isolated mitochondria from WT and GOT2 KO mice. The blot was first probed for GOT2, followed by GDH and porin. For GDH and porin, faint residual GOT2 bands after stripping the blot for GDH and porin can be seen near the 37 kD marker. *B*, quantitative data for GOT2 in mitochondria from KO and WT mice. Data are expressed as the relative density of each of the KO bands to the average density of the WT bands on the individual blots. n = 20, *p* < 0.0001 by two-tailed, unpaired *t* test.

**Figure 2:**
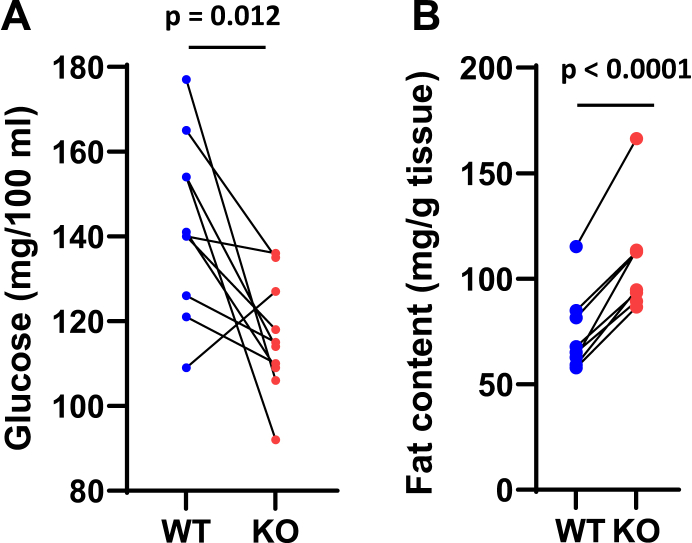
**Six hour fasting glucose and liver fat content in sibling paired WT and GOT2KO mice.***A*, glucose, n = 10. *B*, liver fat content, n = 8. p determined by two-tailed, paired *t* test.

All experiments used KO and WT mice paired by sex, age, and time from the injection of adeno-associated virus (AAV). There was no overt difference in physical appearance or mobility between KO and WT mice. Immunoblotting revealed substantial reduction in GOT2 expression in KO *versus* WT mice (Fig. 1). Since glutamate serves as a substrate for the mitochondrial enzyme GDH as well as GOT2, we also examined GDH expression and found no difference in KO *versus* WT mitochondria. Total body mass as well as fat, lean, and fluid distribution by NMR did not differ between groups (Table 1). Six hour fasting glucose was lower in KO *versus* WT mice while liver fat content was greater in the KO mice by both unpaired and paired comparisons (Table 1 and Fig. 2, respectively).

**Table 1:** Characteristics of 32 pairs of WT and GOT2KO mice

Mice-fed normal chow	WT	GOT2KO	*p*
All mice n (female/male)	32 (18/14)	32 (18/14)	
Age at sacrifice (days)	195 ± 5.6	195 ± 5.7	ns
Time from induction^a^ (days)	79 ± 3.8	79 ± 3.8	ns
Age at induction^a^ (days)	116 ± 3.5	116 ± 3.5	ns
Weight at sacrifice (g)	25.9 ± 0.9	25.4 ± 0.7	ns
Body composition (NMR)			
n (female/male)	22 (11/11)	22 (11/11)	
Total mass (g)	28.5 ± 1.1	28.3 ± 1.0	ns
% fat	20.8 ± 1.2	20.8 ± 1.1	ns
% lean	54.6 ± 1.0	54.5 ± 0.9	ns
% fluid	9.8 ± 0.2	9.9 ± 0.2	ns
6 h fasting glucose (mg/100 ml)	143 ± 7	116 ± 4	0.0032
n (female/male)	10 (4/6)	10 (4/6)	
18h fasted liver fat content (mg/g tissue)	74 ± 7	109 ± 9	0.0093
n (female/male)	8 (6/2)	8 (6/2)	

In all experiments, mice were paired by sex, age, and time after viral AAV injection for the induction of liver-specific GOT2KO or control injection. All except one pair born a day after each other were siblings. Data represent mean ± SE. The 18 h fasting glucose represents values obtained just prior to sacrifice for other experiments.

*p* values determined by two-tailed, unpaired *t* test.

a AAV viral TBG CRE injection for GOT2 KO or AAV viral GFP CRE control.

### GOT2KO alters respiration by liver mitochondria energized with 10 mM succinate + 0.5 mM glutamate at low inner membrane potential

**Figure 3:**
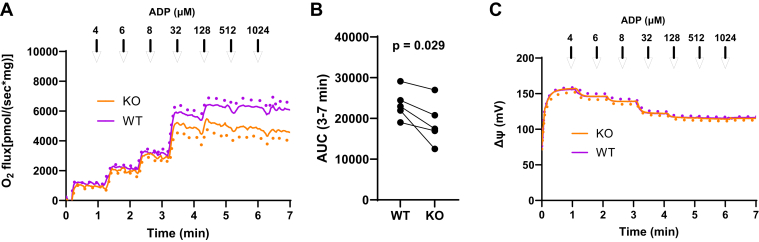
**ADP titrations of isolated liver mitochondria from WT and GOT2KO mice energized by 10 mM succinate + 0.5 mM glutamate.** Liver mitochondria were isolated from five pairs of mice matched for equal age, sex (four female, one male), and time after viral AAV induction of GOT2KO. *A*, oxygen consumption rates (OCR, ± SE shown by *dots*). *B*, area under the curves (AUC) from 3 to 7 min. *p* values determined by two-tailed, paired *t* test. *C*, ΔΨ corresponding to panel measured in three of the five pairs.

Figure 3 shows respiration by liver mitochondria energized with 10 mM succinate + 0.5 mM glutamate (substrate and nitrogen donor for GOT2) isolated from WT and KO mice. As seen in Figure 3*A*, O_2_ flux decreased more at higher ADP (lower ΔΨ) for the KO mice, while Figure 3*B* shows that area under the curve from 3 to 7 min was also lower. ΔΨ did not differ between KO and WT mice (Fig. 3*C*).

### Metabolite accumulation by liver mitochondria energized with 10 mM succinate + 0.5 mM glutamate

**Figure 4:**
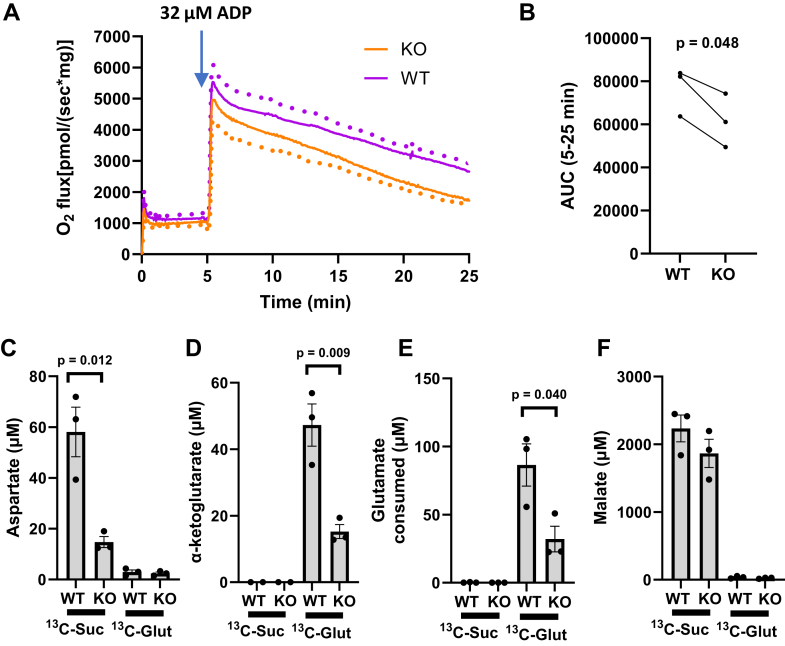
**O_2_ flux and metabolites measured by NMR after incubation of liver mitochondria from WT and GOT2KO mice energized by U^13^C-succinate (10 mM) + unlabeled glutamate (0.5 mM) and repeated using unlabeled succinate (10 mM) + U^13^C-glutamate (0.5 mM).***A*, 175 μg/ml of liver mitochondria from three of the mice utilized in Figure 3 were incubated for 5 min in the absence of ADP before addition of ADP clamped at 32 μM. For each of the three runs, the respiratory values represent the average of the two differentially labeled runs (respiration should be the same independent of how the substrates were labeled). *Dots* indicate SE. *B*, area under the curves of (A) from 5 min to 25 min. *p* value by two-tailed, paired *t* test. *C*–*F*, ^13^C-labeled metabolite levels determined on Oxygraph chamber contents after the incubations of (*A*). Glutamate consumed refers to the difference between the concentration added (500 μM) and the concentration measured by NMR. *p*-values by two-tailed, unpaired *t* test.

Figure 4, *A* and *B* depict O_2_ flux and area under the curves of liver mitochondrial incubations from WT and GOT2KO mice at a clamped ADP concentration of 32 μM, a level at which respiration begins to decrease in the KO mice (Fig. 3*A*). As seen, respiration was lower in the GOT2KO mitochondria over the 20 min period after addition of ADP. Each of these incubations were carried out twice, once energized by U-^13^C-succinate (10 mM) + unlabeled glutamate (0.5 mM) and again energized by unlabeled succinate (10 mM) + U-^13^C-glutamate (0.5 mM) in separate runs. O_2_ flux should not be affected by which substrate was labeled, so each data point for the three repeated determinations represents the average from the two incubations. Metabolite data (Fig. 4, *C* and *D*) show that U-^13^C-succinate labels aspartate but not α-KG while U-^13^C-glutamate labels α-KG and essentially no aspartate (some as natural abundance). These studies can more efficiently be done using a combination of both labels, but the work shown here documents specificity and shows only negligible amounts of unlabeled aspartate and α-KG arising from natural abundance. Again, our 2D NMR method measures only ^13^C-labeled metabolites derived directly from the added ^13^C-labeled energy substrates. As shown in Figure 4, *C* and *E*, aspartate, α-KG, and glutamate consumed (concentration added minus measured) were markedly lower in the KO mitochondria. Malate was nonsignificantly lower (Fig. 4*F*) but consistent with lower respiration. OAA was too low to detect by NMR under the conditions of these experiments. However, the lower aspartate, α-KG, and glutamate consumed in the KO mice imply that the KO reduced the conversion of OAA to aspartate and glutamate to α-KG.

The gradual decrease in O_2_ flux from 5 to 25 min (Fig. 4*A*) was not due to damaged or “exhausted” mitochondria as this decrease could be rapidly reversed by the addition of TMPD (*N,N,N′,N′*-tetramethyl-*p*-phenylenediamine) + ascorbate for downstream electron donation at complex IV (Fig. S1).

### Respiration and metabolite accumulation by WT and GOT2KO liver mitochondria energized by 5 mM succinate + 1 mM glutamate

**Figure 5:**
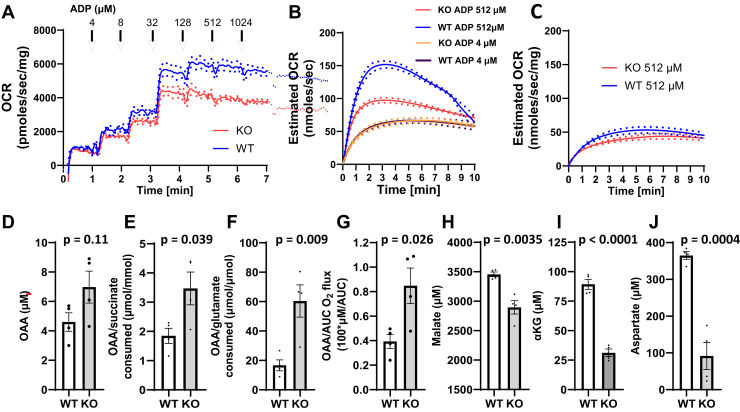
**O_2_ flux and metabolite accumulation by liver mitochondria isolated from WT and GOT2KO mice.***A*, energized by 5 mM succinate + 1 mM glutamate and titrated with incremental increases in the clamped concentrations of ADP in a closed respiratory chamber, n = 2. *B*, energized by 5 mM U^13^C-succinate + 1 mM U^13^C-glutamate and incubated in an open respiratory chamber for 10 min at 4 or 512 μM ADP as indicted, n = 4. *C*, energized by 2 mM U^13^C-Glutamate alone and incubated in an open respiratory chamber for 10 min at 512 μM ADP, n = 2. *Dots* in (*A–C*) indicate SE. *D*–*J*, ^13^C-labeled metabolites measured in the incubation media after the incubations of (B) at 512 μM ADP, n = 4. *p*-values by two-tailed unpaired *t* test.

To assess respiration at different relative levels of succinate and glutamate and to determine if we could detect OAA, we energized mitochondria with U^13^C-succinate (5 mM) + U^13^C-glutamate (1 mM). Titration of GOT2KO mitochondria (175 μg/ml) with ADP in a closed respiratory chamber (Fig. 4*A*) resulted in lower O_2_ flux at higher clamped ADP (lower ΔΨ) consistent with the data in Figure 3*A*, but more strongly manifest. As indicated under “Experimental procedures,” using 175 μg mitochondria/ml, we could not detect OAA. We tried to increase the number of mitochondria but that resulted in rapid loss of O_2_ tension in the respiratory chamber with inadequate time to accurately assess metabolites. Therefore, as described under “Experimental procedures,” we incubated mitochondria in a persistently open to air chamber and quantified O_2_ flux as the difference between measured oxygen concentrations over time and the initial (atmosphere equilibrated) concentration. To clarify, this was only utilized for the experiments in Figure 5, *B* and *C*, not in other experiments within this manuscript.

Figure 5*B* depicts estimated O_2_ flux over 10-min incubations energized with U^13^C-succinate (5 mM) + U^13^C-glutamate (1 mM) for KO and WT mitochondria at low (4 μM) and high (512 μM) clamped ADP. O_2_ flux was markedly lower for the KO mitochondria but only at high ADP (low ΔΨ). Figure 5*C* shows that measuring respiration on 2 mM U^13^C-glutamate alone (twice the concentration as in Fig. 5*B*) by either KO or WT mitochondria at 512 μM ADP resulted in only low O_2_ flux. This indicates that the large part of the O_2_ flux at 512 μM ADP (and the difference between WT and KO O_2_ flux) in panel B is due to succinate (complex II)-energized rather than glutamate-energized respiration.

Figure 5, *D*–*J* show metabolite levels after the open chamber incubations. The results are consistent with the data in Figure 4 but now, importantly, also include data showing higher OAA production by GOT2KO mitochondria and significantly higher OAA production when expressed relative to succinate consumed (substrate generating OAA for GOT2) or glutamate consumed (direct substrate for GOT2) or normalized to respiration itself. The other metabolite results are consistent with Figure 4, *C* and *F*.

### GOT2KO differentially impairs mitochondrial respiration on glutamate versus pyruvate

**Figure 6:**
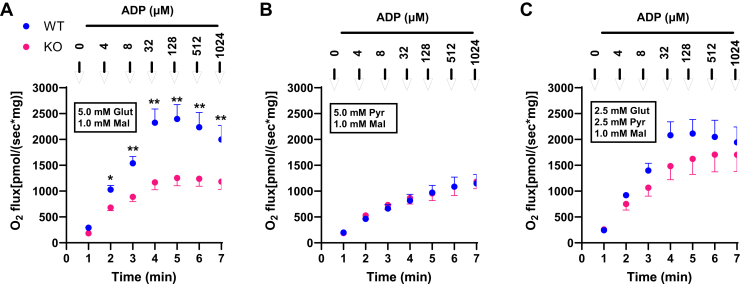
**Differential effect of GOT2KO on liver mitochondrial respiration in a closed Oxygraph chamber energized by malate + glutamate or malate + pyruvate.** Mitochondria were energized by either 5 mM glutamate + 1 mM U^13^C-malate or 5 mM pyruvate + 1 mM U^13^C-malate or a combination as indicated (*boxes*). ADP was incremented at clamped concentrations at the indicated times. *A*, glutamate + malate, n = 4. *B*, pyruvate + malate, n = 4. *C*, combined substrates, n = 3. Data represent mean ± SE. ∗*p* < 0.05, ∗∗*p* < 0.001 by two-factor (genotype x ADP) ANOVA. Mitochondria from the same sibling paired mice were used in all three panels (C examined only three of the four pairs).

Mitochondria were energized by either 5 mM glutamate + 1 mM U-^13^C-malate or 5 mM pyruvate + 1 mM U^13^C-malate (Fig. 6). ADP was incremented at clamped concentrations at the indicated times. Pyruvate and glutamate (with low malate for anaplerosis) are both considered complex I substrates. However, pyruvate drives respiration by generating acetyl-CoA for metabolism through citrate synthase while glutamate drives respiration through generation of α-KG through reactions catalyzed by GOT2 and/or GDH. Therefore, we asked if GOT2KO would differentially affect respiration on pyruvate *versus* glutamate. As shown, this was the case comparing Figure 6, *A* and *B*, with intermediate results for mitochondria incubated on a combination of all substrates (Fig. 6*C*).

**Figure 7:**
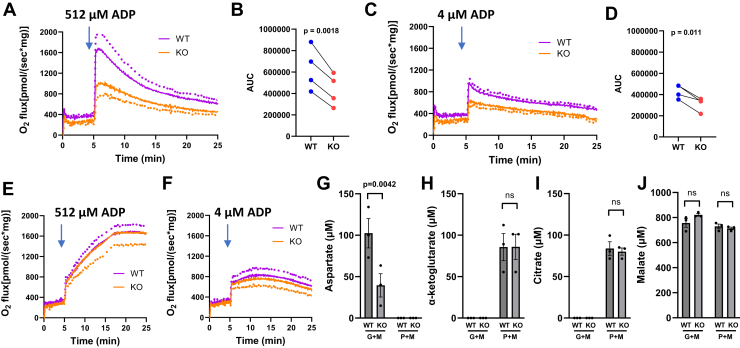
**Differential effect of GOT2KO on mitochondrial respiration energized by either 5 mM glutamate + 1 mM U^13^C-malate or by 5 mM pyruvate + 1 mM U^13^C-malate.** Clamped concentrations of 512 or 4 μM ADP were added at the 5 min time points. *A*, glutamate + U^13^C-malate at 512 μM ADP, n = 4. *B*, area under curves of A. *C*, glutamate + U^13^C-malate at 4 μM ADP, n = 4. *D*, area under curves of C. *E*, pyruvate + U^13^C-malate at 512 μM ADP, n = 4. *F*, pyruvate + U^13^C-malate at 4 μM ADP, n = 4. *G*–*J*, ^13^C-labeled metabolites measured by NMR in three of the four pairs of mice of A. Metabolite measurements were from the chamber contents of the mitochondria incubated in (*A*) and (*E*) energized by unlabeled 5 mM glutamate + 1 mM U^13^C-malate (G + M) at 512 μM ADP or from those incubated in (*E*) energized by unlabeled 5 mM pyruvate + 1 mM U^13^C-malate at 512 μM ADP (P + M). Data represent mean ± SE. *p* values determined by two-tailed, paired *t* test. *Dots* indicate SE.

Figure 7, *A* and *B* show that respiration energized by 5 mM glutamate +1 mM U^13^C-malate was lower in GOT2KO *versus* WT mitochondria after the addition of 512 μM ADP. Figure 7, *C* and *D* show that respiration energized by 5 mM glutamate +1 mM U^13^C-malate at 4 μM ADP was lower than at 512 μM ADP for both KO and WT mitochondria, but again lower in the KO than WT mitochondria. Figure 7, *E* and *F* show that respiration did not differ between KO and WT mitochondria energized by 5 mM pyruvate + 1 mM U^13^C-malate at 512 or 4 μM ADP. In these experiments, the labeled tracer was U^13^C-malate, hence the same label for glutamate or pyruvate studies. Figure 7, *G*–*J* show metabolite levels in the respiratory media after the incubations of panels 7A or 7E. Aspartate, a product of OAA generated from the GOT2 reaction, is decreased by GOT2 knockdown (Fig. 6*G*) but only when energized by glutamate, not by pyruvate. Figure 6*H* shows that α-KG was not detectable in mitochondria energized by glutamate + malate as it is a product of glutamate that was not labeled. In contrast, pyruvate + malate generated α-KG as pyruvate reacts with labeled OAA (from labeled malate) generating labeled citrate and, hence, downstream labeled α-KG. These findings imply that GOT2 KO does not directly affect pyruvate metabolism since levels of α-KG and citrate (Fig. 6, *H* and *I*) were no different for the KO and WT mitochondria. Malate (Fig. 6*J*), of course, was abundantly detected as it was the added labeled substrate.

### GOT2KO did not alter liver mitochondrial respiration on energized by fatty acid oxidation

**Figure 8:**
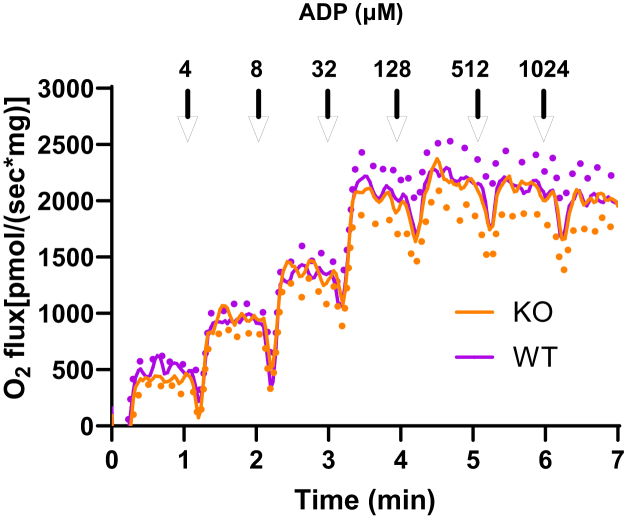
**Lack of a differential effect of GOT2KO on liver mitochondrial respiration energized by fatty acid oxidation at clamped levels of ADP.** Mitochondria of four sibling pairs of GOT2KO and WT mice were energized by 25 μM palmitoyl-L-carnitine + 1 mM malate. ADP was incremented at clamped concentrations as indicated. *Dots* indicate SE.

Figure 8 shows no difference between O_2_ flux energized by 25 μM palmitoyl-L-carnitine + 1 mM malate between GOTKO and WT mice.

### Metabolomic profiles and metabolic pathway assessment

**Figure 9:**
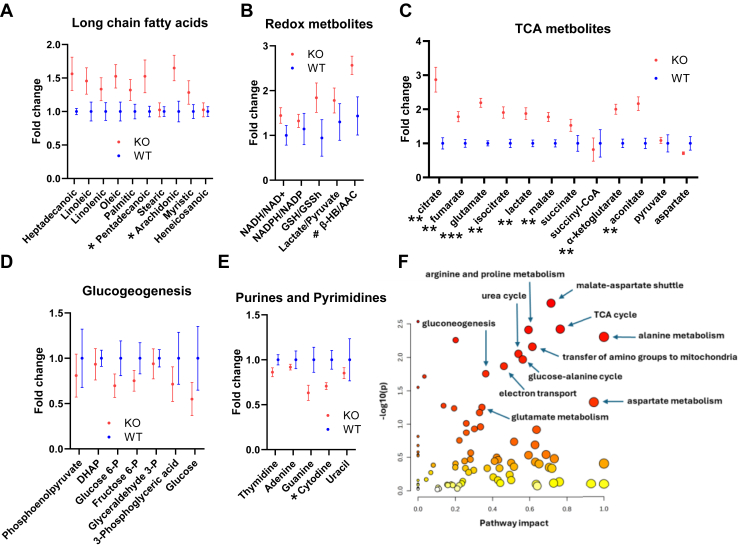
**Specific compounds and pathways based on LC-MS profile of 249 molecules.***A*–*E*, groups of related compounds. *F*, pathway analyses based on entry of the 249 compounds into MetaboAnalyst software. ∗*p* < 0.05, ∗∗*p* < 0.01, ∗∗∗*p* < 0.001, #*p* = 0.05 by 2-tailed, unpaired t-tests uncorrected for multiple comparisons.

A metabolite profile of 249 compounds was obtained on liver tissue from four pairs of overnight (16 h) fasted GOT2KO and WT mice (Fig. 9). The complete profile is listed in supporting information, Table S1. Of note, compounds within related functional groups differed in consistent fashion, although, as individual compounds, several did not differ significantly (Fig. 9, *A–E*). Entering all profile metabolites to MetaboAnalyst revealed overrepresentation of several pathways of interest (Fig. 9*F*).

## Discussion

In past work (5, 6, 7), we found that O_2_ flux in mitochondria of skeletal muscle and brown fat energized at complex II by succinate increased but then decreased on titration with clamped levels of ADP. We provided evidence that the decrease at higher ADP levels was likely due to ΔΨ-dependent accumulation of OAA and OAA inhibition of SDH. Since GOT2 catalyzes the conversion of OAA to aspartate, we hypothesized that GOT2KO would increase OAA concentrations and decrease complex II–energized respiration dependent on ΔΨ.

Our past work did not examine GOT2 effects in muscle or BAT. However, here we decided to first test the above hypothesis in liver mitochondria. OAA effects in liver are of particular importance. The compound is critically positioned in the TCA cycle: 1) to react with acetyl-CoA to form citrate, 2) to act as a reactant in the malate dehydrogenase reaction affecting malate exit from mitochondria, or 3) to generate aspartate and α-KG *via* GOT2. The importance of OAA is clear since hepatic TCA flux underlies multiple metabolic pathways; because malate exit provides OAA for gluconeogenesis; because aspartate is critical to nucleic acid synthesis, the malate-aspartate shuttle, and the urea cycle; and because α-KG leads to downstream TCA flow to succinyl-CoA and hence “short circuits” the classical circular TCA cycle.

Here we investigated the role of GOT2 in regulating liver mitochondrial function as affected under different substrate conditions and under different states of ADP availability and hence membrane potential. Knockout was highly effective based on immunoblotting (Fig. 1). Consistent with our hypothesis regarding GOT2, OAA, and dependence on ΔΨ, we observed significant decreases in respiration by KO compared to WT mitochondria in the presence of glutamate along with a source of OAA, either succinate (Figure 3, Figure 4, Figure 5) or malate (Figs. 6*A* and 7, *A* and *C*). Moreover, we saw no differences in respiration between KO and WT mitochondria when energized by substrates that do not depend on complex II or GOT2 or support a complete TCA cycle needed for the generation of OAA (Figs. 6*B* and 7, *E* and *F*, 8).

Incubation of isolated mitochondria energized by 10 mM succinate plus a low amount of glutamate (0.5 mM) as a substrate for the GOT2 reaction (Fig. 3) showed that respiration decreased in the GOT2KO mitochondria *versus* WT but only at low ΔΨ. O_2_ flux and metabolite data after 20 min of incubation energized in this way at a clamped ADP of 32 μM (Fig. 4) revealed lower respiration in the KO mice (Fig. 4, *A* and *B*). This was associated with marked reductions in aspartate, α-KG, and glutamate consumed suggesting OAA accumulation and inhibition of SDH. Moreover, OAA accumulation and SDH inhibition over time could explain the gradual decrease in O_2_ flux (Fig. 4*A*) but retained capacity to respond to downstream electron donation (Fig. S1). But unfortunately, we could not detect OAA under these conditions. OAA concentrations are very low in liver and are difficult to measure by MS due to instability (8, 9) and could be questioned even when measurements are attempted by mass spectroscopy, although one report suggested a level of < 1 nmol/mg in liver tissue (10), far lower than other TCA metabolites.

As discussed above, increasing the number of mitochondria incubates did not solve the problem measuring OAA due to rapidly dropping oxygen tension. Therefore, we incubated large numbers of mitochondria in a persistently open Oxygraph chamber as well as energizing with more glutamate relative to succinate in order to better drive substrates through GOT2. Figure 5 shows that after 10 min of incubation under the conditions of Figure 5*B*, GOT2KO increased OAA accumulation at high (512 μM) ADP and significantly when normalized to succinate or glutamate consumed or respiration itself (Fig. 5, *D*–*G*). The rationale for this normalization is that OAA accumulation, although enhanced at low ΔΨ, would also be limited by the resultant decrease in respiration (which would also reflect in the consumption of succinate and glutamate). We could not detect OAA when WT or KO mitochondria were incubated at low (4 μM) ADP (as in Figure 5*B*, not shown). We note here that our NMR method is rigidly controlled for stability (11, 12) and can reliably detect OAA down to 1 μM.

As shown in Figure 6, respiration titrated with ADP is differentially regulated by GOT2 dependent on energization by 5 mM glutamate compared to 5 mM pyruvate. In each case, a low concentration of 1 mM malate is added for anaplerotic input. Pyruvate and glutamate (with low malate) are both considered complex I substrates. However, pyruvate drives respiration by generating acetyl-CoA for reaction with OAA to form citrate while glutamate drives respiration through reaction with OAA to generate α-KG and aspartate catalyzed by GOT2. The data show that the effect of GOT2KO is specific for the glutamate reaction with no secondary effect that might alter the citrate synthase reaction.

We also assessed respiration on glutamate + malate or pyruvate + malate over 20 min at clamped ADP concentrations of 512 μM (low potential) or 4 μM (high potential) (Fig. 7). The data again imply that GOT2KO impairs respiration energized by glutamate but not by pyruvate. Metabolites measured by NMR after the incubations energized by glutamate + labeled malate *versus* pyruvate + labeled malate at 512 μM ADP (Fig. 7, *G*-*J*) are as expected, showing that GOT2KO reduced aspartate but had no effect on citrate production from pyruvate.

At the whole mouse level, we observed that GOT2KO did not alter body mass but did increase liver fat. Metabolite profile and pathway data were consistent with these effects. Related to liver fat, profiled compounds included levels in 10 fatty acids of 12 or more carbons. As Figure 9*A* shows, almost all were greater in GOT2KO liver tissue *versus* WT, although for two of the 10, the difference was very small. Moreover, the tissue redox data (Fig. 9*B*) as well as overrepresentation (less operative for GOT2KO because of less aspartate) of the malate aspartate shuttle (Fig. 9*F*) which provides reduced dinucleotides to mitochondria suggest a reduced cytoplasmic dinucleotide milieu consistent with fatty acid synthesis. Note that the term “overrepresentative” in MetaboAnalyst software only indicates a difference between the conditions compared, not in which condition the pathway is most or least operative.

Also, at the whole-mouse level, we observed decreased fasting glucose in the GOT2KO mice *versus* WT. Less metabolites used for gluconeogenesis in the KO liver (Fig. 9*D*) and overrepresentation of the gluconeogenic pathway (Fig. 9*F*, note that overrepresentation as expressed by MetaboAnalyst software would follow from less gluconeogenic metabolites in KO liver) are consistent with reduced fasting glucose. At first thought, one might question the concept that increased liver fat should be associated with decreased fasting glucose and possible decreased gluconeogenesis, since humans with liver steatosis generally have insulin resistance and impaired suppression of hepatic glucose output with increased gluconeogenesis (13). However, we think that the defect in GOT2 and consequent accumulation of OAA would suppress SDH activity leading to decreased generation of malate with less malate to exit from mitochondria. This would result in less cytoplasmic generation of OAA which is needed to initiate gluconeogenesis (14). Of note, oxaloacetate itself does not or poorly crosses the mitochondrial inner membrane (15). We acknowledge that the above is largely speculative and needs confirmation with further study including direct assessment of gluconeogenesis, which is beyond our current focus on mitochondrial function. However, note that Figures 4*F* and 5*H* do show that malate concentrations are reduced associated with increased OAA and decreased respiration in GOT2KO mitochondria *versus* WT incubated under conditions where the complex II energy substrate, succinate, is present.

Additional profiling data of interest included reductions in all four purine and pyrimidine nucleotides as well as uracil (Fig. 9*E*). In this regard, aspartate has been identified as a critical for nucleic acid synthesis (4), serving as a precursor to purines and pyrimidines (16). Hence, the defect in GOT2 might have multiple nonspecific effects leading to hepatic mitochondrial and tissue remodeling.

Finally, our profile data showed that almost all TCA intermediates or compounds that directly supply energy substrates for the TCA cycle were present in greater amounts in KO liver tissue compared with WT, an exception being aspartate whose mitochondrial formation would be impaired by the GOT2 KO (Fig. 9*C*). Higher amounts of TCA substrates do not imply greater TCA activity which depends on enzyme activities. However, we might view the higher substrate amounts in KO liver as an attempt at compensation for otherwise impaired mitochondrial function.

A limitation is that this study was mainly directed at mitochondrial function and metabolite flow. More specific studies are needed regarding how GOT2 affects whole body parameters including direct measurements of gluconeogenesis by tracer analysis and of insulin sensitivity or by assessment of hepatic signaling and gene expression reflecting hepatic glucose and lipid turnover. Moreover, we only examined liver fat in mice-fed normal rodent chow, for example, apart from excess dietary fat. Regarding the NMR method we used to quantify OAA, the assay is sensitive down to 1 μM, equivalent to commercially available kits, which we believe are much less specific especially in the lower range of the linear curves generated by the colorimetric or fluorometric kit methodology. Moreover, in our hands, our methodology takes no more time and costs no more than kit assays and is much less costly when we use NMR to detect multiple metabolites from the same sample (*versus* the use of multiple kits for individual metabolites).

In summary, GOT2KO impairs hepatic mitochondrial respiration energized at complex II. The mechanism, at least in part, includes decreased conversion of OAA to aspartate and accumulation of OAA, a potent inhibitor of SDH and, hence, electron donation at complex II. GOT2KO does not alter hepatic mitochondrial respiration energized by energy substrates primarily acting apart from complex II. Moreover, we provide data suggesting that GOT2KO increases liver fat accumulation and might impair gluconeogenesis, reducing fasting glucose.

## Experimental procedures

### Reagents and supplies

*ADP, 2-*deoxyglucose, U-^13^C-succinate, U-^13^C-glucose, U-^13^C-glutamate, U-^13^C-malate were obtained from Millipore Sigma. Otherwise, reagents, kits, and supplies were as specified or purchased from standard sources.

### Animal procedures

Animals were maintained according to National Institute of Health guidelines and the protocol was approved by our Institutional Animal Care and Use Committee. Male and female C57BL/6J mice (Jackson Laboratories) were fed a normal rodent diet (13% kcal fat diet 7001, Teklad, Envigo) until sacrifice. Mice were euthanized by isoflurane overdose and cardiac puncture.

### Body mass and distribution

Fat, lean, fluid, and body mass were determined by NMR spectrometry using a Bruker mini-spec LF 90II instrument as we have done in the past (17, 18).

### Liver fat content

Fat content was determined by tissue extraction and lipid weight as we have in the past (18).

### Generation of a GOT2 conditional KO mouse

Our institutional Genome Editing Core generated an inducible GOT2 KO mouse on a C57BL/6J background. The second coding exon of *Mus musculus* GOT2 (NM_010325) was flanked by LoxP sites. The deletion of this 157 bp exon resulted in a frame-shift between the first and third exons after Cre-mediated excision. LoxP sites were placed at least 100 bp from the splice acceptor and splice donor sites to protect the splicing mechanisms in the nonexcised allele. CRISPR/Cas9 cut sites with predicted high on-target and low off-target activities were chosen using the CRISPOR algorithm (∗; http://crispor.tefor.net). The target vector was constructed after determining which CRISPR guides cut their target sequence the most efficiently. A CRISPR repair template was made by PCR amplifying the target vector using one phosphorylated primer and one nonphosphorylated primer. The repair template was made single stranded using the “Guide-it Long ssDNA Strandase Kit” from Takara. The GOT2 locus was targeted by microinjecting Cas9:crRNA ribonucleoprotein complex and single stranded repair template into the pronucleus of 0.5-day-old C57BL6/J zygotes. The zygotes were transferred to surrogate females. Pups born were genotyped for the presence of both 5′ and 3′ LoxP sites and the targeted allele completely sequenced.

To induce liver-specific GOT2KO, homozygous floxed mice were injected in the tail vein with AAV CRE (University of Iowa Viral Vector Core, Serotype is AAV2/8) driven by the liver-specific thyroxine-binding globulin promoter. We found marked reduction in liver GOT2 expression after about 6 weeks (Fig. 1).

### Preparation of mitochondria

Mitochondria were prepared by differential centrifugation and purified using a Percoll gradient as we have described (12). All work was performed on ice with equipment cooled to 4 °C. Approximately 0.25 g of freshly harvested liver tissue was placed in 15 ml of cold isolation medium (0.25 M sucrose, 5 mM Hepes [pH 7.2], 0.1 mM EDTA, 0.1% fatty acid-free bovine serum albumin [BSA]) and homogenized for six passes using a Teflon-glass Potter-Elvehjem type tissue grinder in an ice bucket with drill mounted pestle set to 300 rpm. The homogenate was then centrifuged at 500*g* for 10 min. The supernatant was transferred to an Oakridge screw cap tube and spun at 10,000*g* for 10 min to generate a crude pellet. This pellet was resuspended in a solution of three parts Percoll to seven parts isolation medium for centrifugation in a Beckman XL-80 ultracentrifuge with a SW60 swinging-bucket rotor for 30 min at 90,000*g* max. The mitochondrial fraction near the bottom of the tube was transferred to a microfuge tube and diluted with isolation medium lacking BSA. The tube was centrifuged for 10 min at 10,000 x g. The pellet was again resuspended without BSA and the spin repeated. The final pellet was resuspended at 50% (v/v) in isolation medium without BSA. Protein content was determined by the Bradford method. Typical yields were approximately 12 to 15 mg of liver mitochondrial protein per gram of liver.

Mitochondrial integrity was determined by cytochrome C release using a commercial kit (Cytochrome C Oxidase Assay Kit, Millipore-Sigma) documenting 96% intact mitochondria, a value well within an acceptable range (19). In addition, we observed an increase in respiration at high ADP *versus* baseline (*i.e.* near state three *versus* state 4) as large as about 10-fold (Figs. 5 and 6). The ratio of respiratory states (state 3/state 4) is considered a good indicator of mitochondrial functional integrity (20).

### Respiration and membrane potential

All studies of mitochondrial respiration and inner membrane potential utilized freshly isolated and purified mitochondria on the day of the experiments. O_2_ flux was determined using an Oxygraph-2k high resolution respirometer (Oroboros Instruments). When measured, ΔΨ was determined simultaneously with respiration using a potential-sensitive tetraphenylphosphonium electrode fitted into the Oxygraph incubation chamber with a volume of 2 ml. For determination of inner membrane potential, a tetraphenylphosphonium standard curve was performed in each run by adding tetraphenylphosphonium chloride at concentrations of 0.25, 0.5, and 0.75 μM prior to the addition of mitochondria to the chamber. Different amounts of mitochondria (see figure legends) were required for the respirometry studies depending on substrate conditions and whether the organelles were subsequently processed for determination of metabolites by NMR or mass spectrometry. Mitochondria were incubated with stirring at 37 °C in 2 ml of ionic respiratory buffer (105 mM KCl, 10 mM NaCl, 5 mM Na_2_HPO_4_, 2 mM MgCl_2_, 10 mM Hepes pH 7.2, 1 mM EGTA, 0.2% defatted BSA) with 10 U/ml hexokinase (Worthington Biochemical) and 10 mM 2-deoxyglucose (2DOG). When ADP was included in incubations, the concentrations were clamped (see below) at the desired level.

Although the O_2_ tension in closed chamber Oxygraph runs drops with time, the rate of respiration is little affected until O_2_ levels become very low. However, since incubations were carried out for 20 min, it was necessary to periodically open the chamber to prevent marked deterioration in the oxygen content of the medium. A representative Oxygraph tracing depicting this process is shown in Fig. S1. Exporting the data to Excel allowed calculation of expected O_2_ flux during chamber openings based on projected linear changes from opening to closing time points.

### Modified respiratory technique enabling quantification of OAA production

In past work, we found that incubating 175 μg/ml muscle or BAT mitochondria energized by 10 mM succinate, as described above, enabled robust assessment of OAA production in the media after incubation. However, incubation of liver mitochondria in this way resulted in no detectable OAA. To try and detect OAA, we then increased the number of mitochondria incubated. However, that resulted in very rapid depletion of oxygen within the respiratory medium without time to detect metabolites of the added succinate or to adequately re-establish oxygen tension. Therefore, we incubated liver mitochondria with the chamber persistently open to air. By incubating six-fold more mitochondria than typically used, we were able to assess OAA production dependent on ΔΨ. In this way, we could incubate mitochondria for 10 min avoiding loss of O_2_ tension to the point of impaired mitochondrial O_2_ consumption, which is known to be robust down to O_2_ tensions of 20% or less of baseline (atmospheric equilibration).

The Oxygraph calculates and reports the oxygen consumption rate (OCR) assuming a closed respiratory chamber (21). If open, the reported OCR will be inaccurate since the value is calculated from the derivative of the oxygen content which drops over time. However, the electrode does continuously and accurately record the O_2_ concentration. As mitochondria consume oxygen in an open chamber, the media concentration represents the balance between that absorbed and that replaced by the atmosphere. Hence, the OCR over any small time interval is the mean difference between measured and baseline over the time in question. Because there may be some lag time, we take this value as “estimated” OCR. The Oxygraph measures oxygen concentrations every 2 s; hence, we calculated estimated OCR per second, dividing by 2. For calculations, we exported the Oxygraph data to Microsoft Excel. Note that even if we do not know the exact lag time, the data comparing one condition to another should be affected in like-fashion. We recently reported this technique in a method-related manuscript (3). The production of OAA per minute by liver mitochondria appeared approximately equal over five *versus* 10 min (3). Moreover, if there were loss of stability over time of incubation, this should likely affect both conditions compared (*e.g.* WT *versus* KO) in similar fashion.

### ADP recycling and generation of the 2DOG energy clamp

This was done using a method we previously (12, 22) developed to carry out studies of isolated mitochondria under conditions of clamped ADP and membrane potential. Mitochondrial incubations were performed in the presence of hexokinase, excess 2DOG, and varying amounts of added ADP. ATP so generated under these conditions drives the conversion of 2DOG to 2DOG phosphate while regenerating ADP. The reaction occurs rapidly and irreversibly thereby effectively clamping membrane potential determined by available ADP. This was in fact the case as we have demonstrated in the past for rat and mouse muscle (7, 22), mouse liver (23), and mouse heart mitochondria (23).

### Metabolite measurements by NMR

Metabolite measurements were performed as we previously described (7, 11, 24) on the contents of the Oxygraph chamber after mitochondrial incubation with ^13^C-labeled substrates in the same media used for measuring respiration. Immediately after mitochondrial incubations, 1.5 ml of the chamber content was placed in tubes on ice and acidified with 91 μl of 70% perchloric acid. The solutions were then thoroughly mixed, sonicated on ice for 30 s at a power setting of 4 W, and stored at −80 °C for up to 2 weeks. The sample tubes were then thawed on ice and centrifuged at 50,000*g* for 20 min at 4 °C. Supernatants were removed and 10 N KOH was added to bring the pH to 7.4 followed by centrifugation at 16,000*g* for 15 min at 4 °C to remove precipitated salts. The cleared, neutralized supernatants were then stored at −80 °C prior to NMR studies. For NMR sample preparations, 350 μl of the stored supernatant was added to 150 μl of 50 mM sodium phosphate, pH 7.4 in deuterium oxide for metabolite measurement. ^13^C and ^1^H NMR assignments of succinate, malate, fumarate, OAA, citrate, pyruvate, aspartate, glutamate, and α-KG were obtained by using standard compounds. OAA was found to be unstable with a half-life about 14 h when tested at pH 7.4 and temperature at 25 °C. Therefore, after mitochondrial incubation, perchloric acid extraction was carried out as quickly as possible to destroy the mitochondrial enzymes and minimize the degradation of OAA. In addition, for determination of stability, known amounts of OAA were subjected to parallel incubation, perchloric acid extraction, neutralization, and storage.

We examined the effect of sample storage under the acidified condition for OAA, malate, fumarate, α-KG, and pyruvate. No loss of stability was found over one- or two-week time periods (25). Succinate and citrate should be stable when acidified based on their structure. Moreover, even if there were some small effects of storage, that should be equal for samples comparing one condition to another (as samples from each condition for a given experiment were stored side by side over the same period).

This 2-dimensional NMR technique assessed both ^13^ C/^1^H HSQC and HMQC spectra collected at 25 °C on a Bruker Avance II 800 MHz NMR spectrometer equipped with a sensitive cryoprobe for the perchloric acid–extracted samples for quantification of metabolites of the mitochondrial incubations. All NMR spectra were processed using NMRPipe package (26) and analyzed using NMRView (27). Peak heights were used for quantification.

Notably, metabolites measured by the above two-dimensional technique represent the amounts accumulated during respiration and only represent metabolites generated from the labeled substrates used to energize mitochondria. Hence, they represent traced metabolites not confounded by any amounts present before incubation.

### Metabolic profiles

Metabolic profiling was carried out for 249 compounds by liquid chromatography-mass spectrometry. Detailed methodology is listed in supporting information. The raw peak area data were corrected for instrument drift using the Normalization and Evaluation of MS-based Metabolomics Data webtool (28). The corrected values were then ratiometrically normalized by dividing each metabolite's corrected value by the total sum of all corrected metabolite values within the sample. This normalization method was applied uniformly across all metabolites in each sample. For metabolic pathway analysis, the data were analyzed by the MetaboAnalyst software freely available online using the mouse Small Molecule Pathway Database.

### Immunoblot analyses

Mitochondria extracts prepared as above were utilized for immunoblotting. Blots were incubated overnight at 4 °C with affinity-purified antibody to GOT2 (1/1000, Rabbit anti-mouse polyclonal GOT2, Millipore Sigma, #AV43517); GDH (1/1000, Rabbit anti-mouse monoclonal GDH, Cell Signaling technology, # 12793); or cytochrome C (1/500, Rabbit polyclonal cytochrome C, Cell Signaling technology, # 4272). Blots were washed with tris-buffered saline with tween-20 and exposed to anti-rabbit horseradish peroxidase–conjugated secondary antibody (1/10,000). Blots were washed again and developed by enhanced chemiluminescence using a standard kit (ECL Prime, Cytiva). Specificity is implied by the manufacturer of these antibodies and supported by the clean signals only at expected kD values and the absence of signals in tissues wherein the proteins are not expressed (brown fat for GOT2 and adult muscle for GDH).

### Statistics

Data were analyzed by two-tailed, *t* test or two-factor (mixed model) ANOVA with multiple comparisons as indicated in the figure legends using GraphPad Prism (GraphPad Software, Inc.). Significance was considered at *p* < 0.05.

## Data availability

All data are contained within the manuscript and supporting information. Data will be shared upon request by contacting William Sivitz, william-sivitz@uiowa.edu, University of Iowa, Division of Endocrinology and Metabolism, Iowa City IA, 52,246, USA.

## Supporting information

This article contains supporting information (28).

## Conflicts of interest

The authors declare that they have no conflicts of interest with the contents of this article.
