# Supplementary material for: Hepatic glutamic-oxaloacetic transaminase promotes mitochondrial respiration energized at complex II and alters whole body metabolism
Source: J Biol Chem. 2025 May 21;301(6):110261. doi: 10.1016/j.jbc.2025.110261 (PMC12212131; doi:10.1016/j.jbc.2025.110261)
Supplement: Supporting information [file mmc1.pdf]

SUPPORTING INFORMATION

Hepatic glutamic-oxaloacetic transaminase (GOT2) promotes mitochondrial respiration energized at complex II and alters whole body metabolism

Brian D. Fink, Ritu Som, Adam J. Rauckhorst, Eric B. Taylor, Liping Yu, and William I. Sivitz

Contents in order listed

Supplementary methods: LC-MS for metabolite profiling of 249 compounds
Supplementary figure 1
Supplemental table 1
Supplemental table 2

LC-MS for metabolite profiling of 249 compounds:

Sample Processing: Tissue samples were lyophilized and transferred to tubes containing ceramic beads. 18-fold (w/v) extraction solvent (2:2:1 acetonitrile:methanol:water containing a mixture of ^{13}C and ^{2}H (D) labeled internal standards including citric acid (2,2,4,4-D4), succinic acid (2,2,3,3-D4), l-valine (2,3,4,4,4,5,5,5-D8), l-glutamic acid ($^{13}C_5$), l-glutamine ($^{13}C_5$), l-lysine ($^{13}C_6$), l-methionine ($^{13}C_5$), l-serine ($^{13}C_3$), and l-tryptophan ($^{13}C_{11}$)) was added to each sample, and the mixtures were homogenized using an Omni International Bead Ruptor. Homogenized samples were rotated in -20C for 1 hr and then centrifuged at 21,000 g for 15 min. 300 µl of the resulting cleared metabolite extracts were transferred to new 1.5ml microcentrifuge tubes and dried using a speed vac apparatus.

LC-MS Method for HILIC samples: Extracts were dried, reconstituted in 30 µL acetonitrile/water (1:1 v/v) vortexed well, kept in -20C overnight, centrifuged at 21,000 g for 15 min, and the supernatants were transferred to LC-MS autosampler vials for analysis.

LC-MS instrumentation: LC-MS data were acquired on a Thermo Q Exactive hybrid quadrupole Orbitrap mass spectrometer with a Vanquish Flex UHPLC system or Vanquish Horizon UHPLC system.

Liquid Chromatography: The LC column used was a Millipore SeQuant ZIC-pHILIC (2.1 X 150 mm, 5 µm particle size) with a ZIC-pHILIC guard column (20 x 2.1 mm). The injection volume was 2 µL.

Mobile phase is as follows:

Solvent A: 20 mM ammonium carbonate [(NH4)2CO3] and 0.1% Ammonium Hydroxide (v/v) [NH4OH] *[Note: pH is ~9.1]*

Solvent B: Acetonitrile

The method was run at a flow rate of 0.150 mL/min.

The gradient starts at 80% B and decreasing to 20% B over 20 minutes; returning to 80% B in 0.5 minutes; and held there for 7 minutes. (PMID: 28388410).

There is a 2-minute pre-equilibration time prior to sample injection and during re-equilibration from 24.5 to 26.5 minutes flow is increased to 0.3 ml/min.

High Resolution Mass Spectrometer: The mass spectrometer was operated in full-scan, polarity-switching mode from 1 to 20 minutes, with the spray voltage set to 3.0 kV, the heated capillary held at 275 °C, and the HESI probe held at 350 °C. The sheath gas flow was set to 40 units, the auxiliary gas flow was set to 15 units, and the sweep gas flow was set to 1 unit. MS data acquisition was performed in a range of m/z 70–1,000, with the resolution set at 70,000, the AGC target at 1 × 106, and the maximum injection time at 200 ms.(PMID: 28388410)

Data Analysis: Acquired LC-MS data were processed by Thermo Scientific TraceFinder 4.1, 5.1, and 5.2 software, and metabolites were identified based on the University of Iowa Metabolomics Core facility standard-confirmed, inhouse library. The raw peak area data were corrected for instrument drift using the Normalization and Evaluation of MS-based Metabolomics Data webtool (PMID 28525573). The corrected values were then total ratiometrically normalized by dividing each metabolite's corrected value by the total sum of all corrected metabolite values within the sample. This normalization method was applied uniformly across all metabolites in each sample. Normalized data were expressed relative to a value of 1.0 in WT liver.

LC-MS for metabolite profiling of 249 compounds (continued):

Tissue extracts were prepared using a defined ratio of 40 μL extraction solvent per 1 mg of tissue, which ensured comparable extract concentrations and consistent LC-MS sample loading across all samples. While tissue weight-based normalization would indeed be more suitable for absolute quantification, particularly in the context of metabolites such as fatty acids, the aim of our study was to assess relative differences in metabolite levels between WT and GOT2 KO livers. This relative approach aligns with comparisons presented in this analysis and in our experience routinely outperforms sample mass/volume-based normalization on a CV/%relative standard deviation basis.

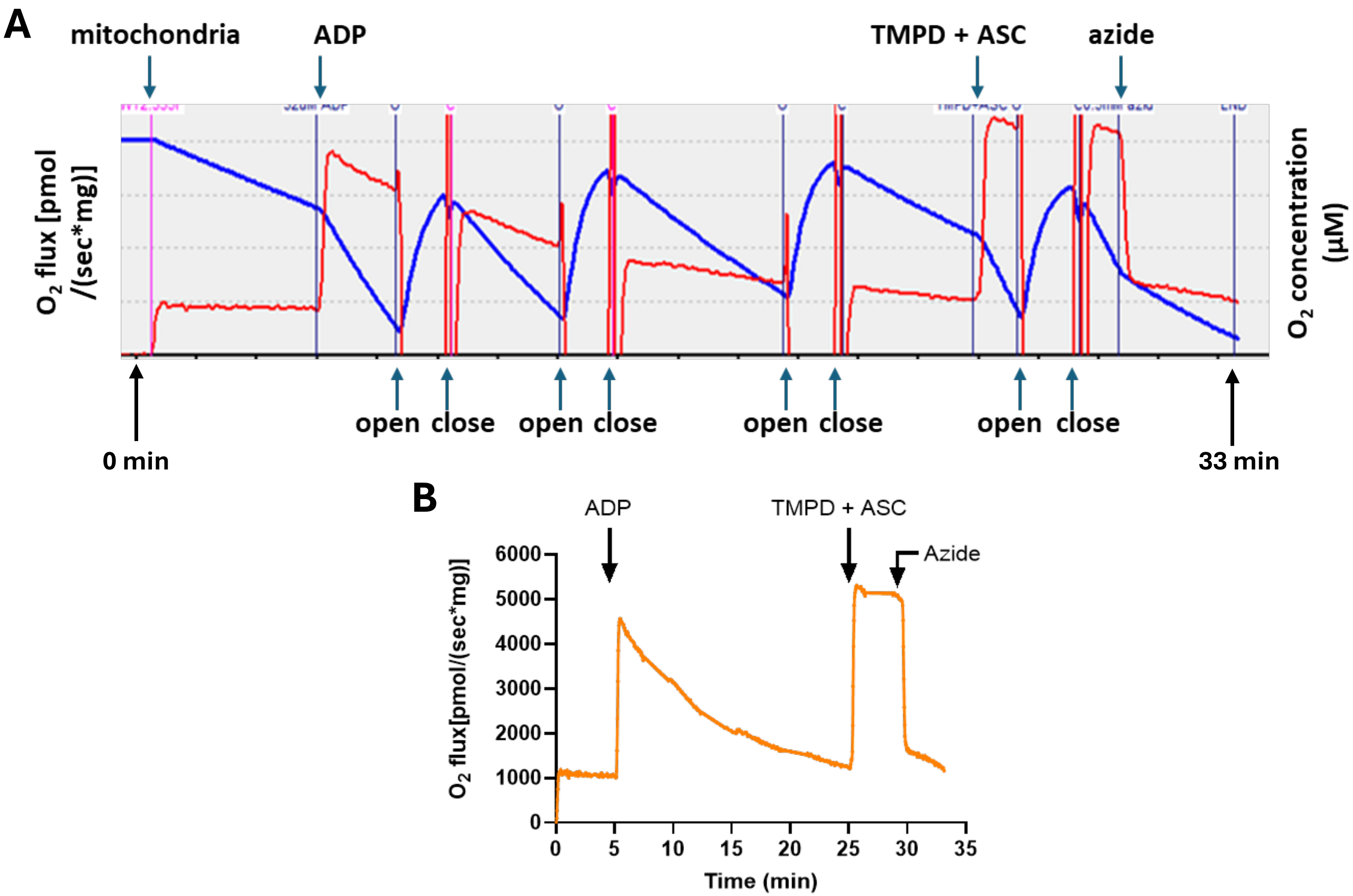

Supplemental figure 1. Liver mitochondrial respiration and effect of TMPD + Ascorbate. A) Copy of Oxygraph computer output. Mitochondria (175 µg/ml) were energized by 10 mM succinate + 0.5 mM glutamate at a clamped ADP concentration of 32 µM. Arrows indicate times during which the respiratory chamber was open to air to re-establish oxygen tension. 0.5 mM TMPD and 2 mM ascorbate (ASC) followed by 0.5 mM sodium azide were added as shown. The red curve represents O_2 flux. The blue curve represents O_2 concentration. B) Data as shown after import into Excel for calculation and insertion of expected values during periods of open chamber.

Supplemental table 1. 249 metabolites measured by LC-MS, fold change, and p-values (2-tailed, unpaired and paired t-tests uncorrected for multiple caparisons)

	Mean KO	SE KO	Mean WT	SE WT	Fold chance KO/WT	p (unpaired t)	p (paired t)
2',3'-cyclic AMP	0.916	0.151	1.000	0.327	0.916	0.822	0.854
2',3'-cyclic CMP	0.307	0.067	1.000	0.618	0.307	0.308	0.327
2-Hydroxybutyric acid	2.386	0.420	1.000	0.181	2.386	0.023	0.026
2-Hydroxyglutaric acid	1.004	0.085	1.000	0.038	1.004	0.966	0.972
2-Methylcitric acid	0.466	0.071	1.000	0.122	0.466	0.009	0.034
3-Hydroxyisobutyric acid	0.893	0.062	1.000	0.041	0.893	0.199	0.265
3-Methylhistamine	0.622	0.082	1.000	0.515	0.622	0.496	0.518
3-Phosphoglyceric acid	0.714	0.191	1.000	0.287	0.714	0.438	0.579
4-Hydroxy-2-nonenal	0.807	0.118	1.000	0.185	0.807	0.413	0.436
4-Hydroxyphenylpyruvic acid	0.866	0.137	1.000	0.208	0.866	0.608	0.707
4-Hydroxyproline	0.649	0.107	1.000	0.343	0.649	0.366	0.361
4-Methylcatechol	1.578	0.038	1.000	0.339	1.578	0.141	0.178
5-HIAA	1.070	0.164	1.000	0.185	1.070	0.788	0.572
5-Methylthioadenosine	0.945	0.071	1.000	0.144	0.945	0.745	0.791
6-Phosphogluconic acid	1.217	0.078	1.000	0.076	1.217	0.093	0.190
Acetoacetic acid	0.934	0.141	1.000	0.343	0.934	0.865	0.867
Acetyl-CoA	0.531	0.210	1.000	0.297	0.531	0.244	0.367
Acetyllysine	1.077	0.196	1.000	0.107	1.077	0.743	0.491
Aconitic acid	2.166	0.200	1.000	0.151	2.166	0.004	0.031
Adenine	0.919	0.032	1.000	0.098	0.919	0.462	0.492
Adenine deoxyribonucleoside	1.323	0.109	1.000	0.112	1.323	0.084	0.152
Adenosine	1.020	0.080	1.000	0.266	1.020	0.945	0.939
Adenosine diphosphate ribose	1.511	0.281	1.000	0.123	1.511	0.147	0.223
Adenylsuccinic acid	1.747	0.567	1.000	0.465	1.747	0.348	0.459
ADP	0.969	0.081	1.000	0.031	0.969	0.730	0.785
ADP-ribose 1"-2" cyclic phosphate	1.598	0.316	1.000	0.102	1.598	0.121	0.179
Alanine	1.084	0.076	1.000	0.068	1.084	0.440	0.559
ALCAR	1.077	0.040	1.000	0.100	1.077	0.504	0.606
Allantoin	0.821	0.072	1.000	0.152	0.821	0.329	0.255
L-Allothreonine	0.794	0.096	1.000	0.218	0.794	0.420	0.213
Alpha-ketoglutaramate	1.012	0.056	1.000	0.157	1.012	0.943	0.949
Alpha-ketoglutarate	2.002	0.147	1.000	0.124	2.002	0.002	0.033
alpha-ketoisocaproic acid	1.185	0.135	1.000	0.235	1.185	0.520	0.226
alpha-ketoisovaleric acid	1.182	0.146	1.000	0.349	1.182	0.648	0.604
Aminoacetone	0.657	0.066	1.000	0.145	0.657	0.075	0.202
Aminoadipic acid	1.929	0.613	1.000	0.260	1.929	0.212	0.287
Aminolevulinic acid	0.649	0.107	1.000	0.343	0.649	0.366	0.361
AMP	1.240	0.115	1.000	0.205	1.240	0.348	0.423
Anserine	1.037	0.228	1.000	0.218	1.037	0.910	0.845
Arachidonic acid	1.648	0.189	1.000	0.154	1.648	0.038	0.135
Arginine	0.917	0.118	1.000	0.126	0.917	0.649	0.751

Argininosuccinic acid	1.739	0.258	1.000	0.229	1.739	0.076	0.044
Ascorbic acid	1.868	1.122	1.000	0.592	1.868	0.519	0.403
Asparagine	0.815	0.094	1.000	0.067	0.815	0.160	0.213
Aspartic acid	0.712	0.041	1.000	0.200	0.712	0.206	0.279
ATP	1.026	0.152	1.000	0.137	1.026	0.902	0.923
Azelaic acid	0.617	0.171	1.000	0.268	0.617	0.273	0.259
Benzoic acid	0.944	0.094	1.000	0.094	0.944	0.686	0.083
beta-Alanine	1.061	0.055	1.000	0.130	1.061	0.678	0.539
Betaine	1.061	0.054	1.000	0.103	1.061	0.623	0.499
Butanoic acid	0.936	0.041	1.000	0.066	0.936	0.442	0.430
Butyryl-CoA	0.570	0.166	1.000	0.330	0.570	0.288	0.429
cAMP	0.931	0.168	1.000	0.295	0.931	0.846	0.875
Capric acid	0.969	0.058	1.000	0.106	0.969	0.809	0.703
Caproic acid	0.861	0.053	1.000	0.086	0.861	0.220	0.312
Caprylic acid	0.978	0.011	1.000	0.174	0.978	0.902	0.905
Decanoylcarnitine	1.227	0.369	1.000	0.299	1.227	0.650	0.609
Dodecanoylcarnitine	1.205	0.312	1.000	0.313	1.205	0.659	0.613
Tetradecanoylcarnitine	1.062	0.190	1.000	0.307	1.062	0.869	0.835
L-Palmitoylcarnitine	1.000	0.091	1.000	0.204	1.000	1.000	0.999
Stearoylcarnitine	1.231	0.049	1.000	0.171	1.231	0.243	0.305
oleoyl-L-carnitine	1.168	0.172	1.000	0.177	1.168	0.523	0.449
arachidonoyl-L-carnitine	2.173	0.328	1.000	0.180	2.173	0.020	0.081
Propionylcarnitine	1.030	0.160	1.000	0.089	1.030	0.874	0.863
butyryl-L-carnitine	1.092	0.083	1.000	0.136	1.092	0.587	0.623
Isovalerylcarnitine	1.055	0.137	1.000	0.254	1.055	0.854	0.858
Hexanoylcarnitine	0.969	0.281	1.000	0.398	0.969	0.952	0.949
Octanoylcarnitine	1.190	0.374	1.000	0.332	1.190	0.718	0.686
glutarylcarnitine	0.874	0.146	1.000	0.053	0.874	0.450	0.451
Carnitine	1.315	0.084	1.000	0.092	1.315	0.044	0.019
Carnosine	1.133	0.230	1.000	0.116	1.133	0.623	0.669
CDP	1.166	0.085	1.000	0.061	1.166	0.165	0.198
CDP-Ethanolamine	0.664	0.046	1.000	0.130	0.664	0.050	0.123
CDP-Ribitol	0.990	0.141	1.000	0.116	0.990	0.957	0.942
Cholic acid	2.739	0.663	1.000	0.273	2.739	0.052	0.055
Choline	1.271	0.253	1.000	0.235	1.271	0.462	0.588
Citicoline	0.591	0.069	1.000	0.242	0.591	0.154	0.115
Citraconic acid	0.918	0.079	1.000	0.050	0.918	0.414	0.559
Citramalic acid	1.004	0.085	1.000	0.038	1.004	0.966	0.972
Citric acid	2.867	0.364	1.000	0.166	2.867	0.003	0.024
Citrulline	0.990	0.110	1.000	0.194	0.990	0.965	0.959
CMP	1.310	0.171	1.000	0.110	1.310	0.178	0.274
CoA	1.478	0.171	1.000	0.420	1.478	0.332	0.460
Cortisol	1.055	0.242	1.000	0.241	1.055	0.877	0.915
Creatine	0.992	0.062	1.000	0.035	0.992	0.913	0.905
Creatinine	0.879	0.089	1.000	0.024	0.879	0.238	0.259

CTP	1.177	0.226	1.000	0.213	1.177	0.589	0.644
Cystathionine	1.168	0.242	1.000	0.268	1.168	0.658	0.617
Cytidine	0.538	0.046	1.000	0.104	0.538	0.007	0.010
Cytosine	0.707	0.038	1.000	0.105	0.707	0.039	0.071
dCMP	0.962	0.102	1.000	0.158	0.962	0.846	0.808
Dehydroascorbic acid	2.166	0.200	1.000	0.151	2.166	0.004	0.031
dGTP	1.028	0.170	1.000	0.152	1.028	0.905	0.922
DHAP	0.934	0.173	1.000	0.090	0.934	0.746	0.772
Dihydrobiopterin	1.084	0.067	1.000	0.151	1.084	0.631	0.477
Dihydrofolic acid	0.970	0.562	1.000	0.253	0.970	0.963	0.967
Dimethylarginine	1.349	0.204	1.000	0.082	1.349	0.164	0.175
3,4-Dihydroxybenzeneacetic acid	0.923	0.123	1.000	0.068	0.923	0.601	0.478
dTDP-D-glucose	0.806	0.078	1.000	0.059	0.806	0.094	0.154
FAD	1.022	0.028	1.000	0.088	1.022	0.819	0.822
Fructose 2,6-bisphosphate	0.716	0.159	1.000	0.185	0.716	0.288	0.213
Fructose 6-phosphate	0.752	0.115	1.000	0.172	0.752	0.275	0.043
Fumaric acid	1.783	0.149	1.000	0.116	1.783	0.006	0.037
Galacturonic acid	0.924	0.073	1.000	0.070	0.924	0.480	0.624
gamma-Glutamylcysteine	1.893	0.504	1.000	0.354	1.893	0.198	0.202
GDP	0.866	0.084	1.000	0.114	0.866	0.379	0.495
Glucaric acid	0.841	0.128	1.000	0.138	0.841	0.432	0.559
Gluconic acid	1.045	0.075	1.000	0.021	1.045	0.584	0.665
Glucosamine 6-phosphate	0.886	0.103	1.000	0.184	0.886	0.608	0.673
Glucose	0.550	0.183	1.000	0.352	0.550	0.299	0.383
Glucose 6-phosphate	0.697	0.130	1.000	0.191	0.697	0.238	0.039
Glucuronic acid	0.924	0.073	1.000	0.070	0.924	0.480	0.624
Glutamic acid	2.191	0.132	1.000	0.074	2.191	0.000	0.001
Glutamine	1.032	0.059	1.000	0.142	1.032	0.843	0.880
Glyceraldehyde 3-phosphate	0.939	0.165	1.000	0.096	0.939	0.760	0.735
Glyceraldehyde 3-phosphate diethyl acetal	0.850	0.067	1.000	0.251	0.850	0.585	0.669
Glyceric acid	0.774	0.125	1.000	0.144	0.774	0.280	0.213
Glycerol	1.326	0.032	1.000	0.059	1.326	0.003	0.033
Glycerol 3-phosphate	1.168	0.055	1.000	0.170	1.168	0.383	0.458
Glycine	0.755	0.045	1.000	0.079	0.755	0.035	0.124
Glycolic acid	0.909	0.131	1.000	0.089	0.909	0.588	0.700
GMP	1.479	0.257	1.000	0.265	1.479	0.242	0.399
GSH	1.831	0.293	1.000	0.457	1.831	0.177	0.208
GSSG	1.005	0.052	1.000	0.060	1.005	0.950	0.963
GTP	0.958	0.161	1.000	0.159	0.958	0.858	0.900
Guanidinosuccinic acid	0.565	0.395	1.000	0.704	0.565	0.609	0.264
Guanidoacetic acid	0.948	0.234	1.000	0.300	0.948	0.896	0.913
Guanine	0.632	0.085	1.000	0.140	0.632	0.066	0.167
Guanosine	0.828	0.077	1.000	0.115	0.828	0.260	0.292
Heneicosanoic acid	1.025	0.104	1.000	0.073	1.025	0.853	0.874
Heptadecanoic acid	1.563	0.248	1.000	0.046	1.563	0.067	0.105

Heptylic acid	0.778	0.115	1.000	0.058	0.778	0.135	0.108
Hippuric acid	0.925	0.074	1.000	0.052	0.925	0.441	0.497
Histamine	0.324	0.095	1.000	0.786	0.324	0.426	0.432
Histidine	1.056	0.064	1.000	0.039	1.056	0.482	0.600
3-Hydroxy-3-methylglutaryl-CoA	0.600	0.189	1.000	0.126	0.600	0.128	0.116
Homoserine	0.827	0.137	1.000	0.189	0.827	0.487	0.114
Homovanillic acid	0.915	0.075	1.000	0.144	0.915	0.619	0.626
Hydroorotic acid	0.721	0.073	1.000	0.258	0.721	0.338	0.339
Hydroxyphenyllactic acid	0.915	0.075	1.000	0.144	0.915	0.619	0.626
Hypotaurine	0.872	0.220	1.000	0.420	0.872	0.796	0.810
Hypoxanthine	2.414	0.677	1.000	0.489	2.414	0.141	0.268
Imidazoleacetic acid	1.045	0.135	1.000	0.173	1.045	0.844	0.871
IMP	1.003	0.245	1.000	0.637	1.003	0.997	0.997
Indole	0.817	0.127	1.000	0.279	0.817	0.572	0.583
Indole-3-carboxaldehyde	0.785	0.120	1.000	0.029	0.785	0.133	0.115
Indole-3-lactic acid	0.815	0.231	1.000	0.181	0.815	0.553	0.530
Indoxyl sulfate	0.901	0.349	1.000	0.260	0.901	0.828	0.786
Inosine	2.067	0.719	1.000	0.660	2.067	0.316	0.438
Isocitrate	1.906	0.166	1.000	0.122	1.906	0.005	0.040
Isoleucine	0.712	0.161	1.000	0.071	0.712	0.153	0.077
Itaconic acid	0.996	0.074	1.000	0.090	0.996	0.974	0.981
Kynurenine	0.926	0.170	1.000	0.081	0.926	0.709	0.749
L-allothreonine	0.794	0.096	1.000	0.218	0.794	0.420	0.213
Lactic acid	1.874	0.171	1.000	0.099	1.874	0.004	0.012
Lauric acid	1.087	0.159	1.000	0.100	1.087	0.662	0.259
Leucine	0.784	0.112	1.000	0.106	0.784	0.209	0.013
Linoleic acid	1.456	0.197	1.000	0.141	1.456	0.108	0.222
Linolenic acid	1.334	0.171	1.000	0.132	1.334	0.174	0.222
Lysine	0.955	0.021	1.000	0.109	0.955	0.697	0.670
Malic acid	1.775	0.133	1.000	0.105	1.775	0.004	0.029
Malondialdehyde	0.998	0.061	1.000	0.179	0.998	0.990	0.987
Methionine	0.688	0.103	1.000	0.089	0.688	0.061	0.197
Methylimidazoleacetate	1.260	0.274	1.000	0.111	1.260	0.413	0.217
Methylmalonic acid	1.527	0.177	1.000	0.232	1.527	0.121	0.169
Myristic acid	1.283	0.174	1.000	0.104	1.283	0.212	0.214
N-Acetylasparagine	1.109	0.072	1.000	0.126	1.109	0.484	0.213
N-Acetylaspartic acid	0.732	0.094	1.000	0.110	0.732	0.114	0.240
N-Acetylcysteine	1.599	0.396	1.000	0.451	1.599	0.357	0.493
N-Acetylglutamic acid	1.149	0.141	1.000	0.150	1.149	0.496	0.591
N-Acetylglycine	0.804	0.092	1.000	0.135	0.804	0.275	0.209
N-Acetylmethionine	1.362	0.219	1.000	0.101	1.362	0.183	0.065
N-Acetylneuraminic acid	0.756	0.158	1.000	0.070	0.756	0.209	0.160
N-Acetylproline	0.888	0.105	1.000	0.160	0.888	0.579	0.579
N-Acetylserotonin	1.015	0.072	1.000	0.108	1.015	0.912	0.938
NAD+	0.873	0.101	1.000	0.143	0.873	0.495	0.415

NADH	1.237	0.174	1.000	0.260	1.237	0.477	0.253
NADP+	0.808	0.120	1.000	0.162	0.808	0.378	0.154
NADPH	1.019	0.071	1.000	0.253	1.019	0.945	0.952
2-formamidoacetic acid	0.662	0.046	1.000	0.173	0.662	0.108	0.192
N-Formylmethionine	1.392	0.146	1.000	0.139	1.392	0.099	0.088
Niacin	1.507	0.092	1.000	0.056	1.507	0.003	0.025
Nicotinamide	1.529	0.128	1.000	0.077	1.529	0.012	0.049
Nicotinamide riboside	1.012	0.027	1.000	0.076	1.012	0.885	0.911
N1-methyl-4-pyridone-3-carboxamide	0.963	0.135	1.000	0.087	0.963	0.824	0.600
Nicotinamide ribotide	1.153	0.083	1.000	0.093	1.153	0.264	0.424
Nonanoic acid	0.864	0.123	1.000	0.105	0.864	0.431	0.569
Norvaline	1.061	0.054	1.000	0.103	1.061	0.623	0.499
Oleic acid	1.525	0.175	1.000	0.141	1.525	0.058	0.150
O-Phosphorylethanolamine	0.876	0.061	1.000	0.103	0.876	0.340	0.302
Ophthalmic acid	1.439	0.156	1.000	0.192	1.439	0.126	0.033
Ornithine	1.152	0.028	1.000	0.109	1.152	0.228	0.178
Orotic acid	0.675	0.026	1.000	0.332	0.675	0.367	0.404
Oxalic acid	1.029	0.139	1.000	0.128	1.029	0.883	0.876
Palmitic acid	1.321	0.157	1.000	0.109	1.321	0.144	0.235
Pantothenic acid	1.077	0.117	1.000	0.241	1.077	0.783	0.753
PAP	0.969	0.081	1.000	0.031	0.969	0.730	0.785
Pentadecanoic acid	1.525	0.244	1.000	0.080	1.525	0.086	0.156
Pentanoic acid	0.754	0.028	1.000	0.069	0.754	0.016	0.081
Phenylalanine	0.863	0.077	1.000	0.055	0.863	0.199	0.013
Phosphocreatine	0.798	0.112	1.000	0.246	0.798	0.483	0.299
Phosphoenolpyruvic acid	0.809	0.237	1.000	0.322	0.809	0.649	0.748
Pimelic acid	0.822	0.053	1.000	0.179	0.822	0.377	0.428
Pipecolic acid	1.075	0.369	1.000	0.341	1.075	0.887	0.200
Proline	1.020	0.066	1.000	0.114	1.020	0.886	0.863
Propionic acid	1.031	0.172	1.000	0.254	1.031	0.923	0.782
Pterin	0.856	0.118	1.000	0.066	0.856	0.330	0.287
Pyridoxamine	0.638	0.127	1.000	0.182	0.638	0.154	0.085
Pyroglutamic acid	1.040	0.078	1.000	0.112	1.040	0.778	0.814
Pyruvic acid	1.087	0.089	1.000	0.251	1.087	0.756	0.774
Quinolinic acid	1.497	0.334	1.000	0.105	1.497	0.205	0.157
Raffinose	1.942	0.190	1.000	0.208	1.942	0.016	0.070
Riboflavin	0.872	0.089	1.000	0.129	0.872	0.444	0.394
Ribose 5-phosphate	0.983	0.073	1.000	0.377	0.983	0.965	0.968
Ribulose 5-phosphate	0.882	0.084	1.000	0.068	0.882	0.314	0.294
S-Adenosylhomocysteine	0.964	0.110	1.000	0.134	0.964	0.843	0.744
S-Adenosylmethionine	0.788	0.065	1.000	0.152	0.788	0.248	0.127
Sarcosine	1.077	0.130	1.000	0.115	1.077	0.672	0.724
Sedoheptulose 7-phosphate	0.671	0.118	1.000	0.256	0.671	0.287	0.113
Serine	0.890	0.077	1.000	0.087	0.890	0.380	0.369
Serotonin	1.112	0.256	1.000	0.145	1.112	0.717	0.650

sn-Glycerol 3-phosphate	1.168	0.055	1.000	0.170	1.168	0.383	0.458
Stachyose	2.394	0.201	1.000	0.456	2.394	0.031	0.015
Stearic acid	1.025	0.104	1.000	0.073	1.025	0.853	0.874
Succinic acid	1.529	0.177	1.000	0.232	1.529	0.120	0.171
Succinyl-CoA	0.819	0.341	1.000	0.401	0.819	0.742	0.598
Taurine	0.925	0.063	1.000	0.068	0.925	0.448	0.472
Threonine	0.827	0.137	1.000	0.189	0.827	0.487	0.114
Thymidine	0.861	0.049	1.000	0.057	0.861	0.114	0.006
Thymine	0.917	0.035	1.000	0.038	0.917	0.158	0.055
TMP	0.903	0.089	1.000	0.254	0.903	0.732	0.733
Trigonelline	0.971	0.064	1.000	0.161	0.971	0.874	0.897
Tryptophan	0.781	0.141	1.000	0.089	0.781	0.237	0.133
Tyrosine	0.781	0.140	1.000	0.066	0.781	0.206	0.357
UDP	0.865	0.062	1.000	0.068	0.865	0.189	0.279
UDP-GlcNAc	0.842	0.096	1.000	0.055	0.842	0.204	0.345
UDP-glucose	0.954	0.114	1.000	0.112	0.954	0.780	0.824
UDP-glucuronic acid	1.044	0.149	1.000	0.252	1.044	0.885	0.782
UMP	1.135	0.154	1.000	0.237	1.135	0.649	0.735
Undecanoic acid	1.028	0.190	1.000	0.275	1.028	0.935	0.951
Uracil	0.852	0.060	1.000	0.234	0.852	0.562	0.645
Ureidopropionic acid	1.176	0.247	1.000	0.302	1.176	0.669	0.417
Uric acid	0.490	0.087	1.000	0.576	0.490	0.415	0.438
Uridine	0.850	0.067	1.000	0.251	0.850	0.585	0.669
Urocanic acid	0.982	0.127	1.000	0.202	0.982	0.941	0.939
UTP	0.861	0.125	1.000	0.179	0.861	0.546	0.579
Valine	1.061	0.054	1.000	0.103	1.061	0.623	0.499
Xanthine	1.457	0.194	1.000	0.529	1.457	0.449	0.541
Xanthosine	0.480	0.093	1.000	0.584	0.480	0.413	0.403

Supplementary table 2. Pathway Analysis

Pathway Name	p	-log(p)	Holm p	FDR	Impact
Malate-Aspartate Shuttle	0.002	2.813	0.14294	0.049	0.714
Carnitine Synthesis	0.003	2.535	0.26563	0.060	0.000
Citric Acid Cycle	0.004	2.424	0.33895	0.060	0.764
Arginine and Proline Metabolism	0.004	2.414	0.34331	0.060	0.593
Alanine Metabolism	0.005	2.310	0.43082	0.065	1.000
Ammonia Recycling	0.005	2.260	0.47846	0.065	0.202
Transfer of Acetyl Groups into Mitochondria	0.007	2.161	0.59379	0.072	0.614
Urea Cycle	0.009	2.054	0.75077	0.083	0.539
Glucose-Alanine Cycle	0.011	1.970	0.90004	0.092	0.563
Mitochondrial Electron Transport Chain	0.014	1.870	1	0.106	0.461
Gluconeogenesis	0.017	1.757	1	0.122	0.363
Galactose Metabolism	0.019	1.720	1	0.122	0.034
Phytanic Acid Peroxisomal Oxidation	0.028	1.553	1	0.155	0.000
Aspartate Metabolism	0.046	1.333	1	0.243	0.946
Alpha Linolenic Acid and Linoleic Acid Metabolism	0.053	1.275	1	0.260	0.137
Glutamate Metabolism	0.056	1.254	1	0.260	0.343
Bile Acid Biosynthesis	0.058	1.237	1	0.260	0.194
Lysine Degradation	0.067	1.172	1	0.288	0.330
Glycine and Serine Metabolism	0.097	1.014	1	0.379	0.259
Valine, Leucine and Isoleucine Degradation	0.109	0.962	1	0.411	0.335
Arachidonic Acid Metabolism	0.118	0.927	1	0.422	0.302
Beta-Alanine Metabolism	0.121	0.917	1	0.422	0.636
Nicotinate and Nicotinamide Metabolism	0.133	0.878	1	0.445	0.259
Vitamin B6 Metabolism	0.155	0.810	1	0.503	0.000
Tyrosine Metabolism	0.177	0.751	1	0.556	0.222
Pyruvate Metabolism	0.185	0.732	1	0.562	0.516
Glutathione Metabolism	0.206	0.686	1	0.600	0.628
Pyruvaldehyde Degradation	0.211	0.676	1	0.600	0.000
Plasmalogen Synthesis	0.263	0.580	1	0.724	0.000
Purine Metabolism	0.271	0.567	1	0.724	0.283
Fatty Acid Metabolism	0.288	0.541	1	0.724	0.688
Selenoamino Acid Metabolism	0.291	0.536	1	0.724	0.000
Beta Oxidation of Very Long Chain Fatty Acids	0.294	0.532	1	0.724	0.594
Catecholamine Biosynthesis	0.300	0.522	1	0.724	0.000
Mitochondrial Beta-Oxidation of Long Chain Saturated Fatty Acids	0.317	0.499	1	0.745	0.422
Phosphatidylcholine Biosynthesis	0.332	0.479	1	0.748	0.732
Steroid Biosynthesis	0.340	0.469	1	0.748	0.281
Phosphatidylinositol Phosphate Metabolism	0.343	0.464	1	0.748	0.431
Caffeine Metabolism	0.360	0.443	1	0.748	0.250
Fatty Acid Elongation In Mitochondria	0.362	0.442	1	0.748	0.411
Mitochondrial Beta-Oxidation of Short Chain Saturated Fatty Acids	0.373	0.429	1	0.748	0.630
Phenylacetate Metabolism	0.382	0.418	1	0.748	0.500
Phosphatidylethanolamine Biosynthesis	0.387	0.412	1	0.748	1.000
Fatty Acid Biosynthesis	0.396	0.403	1	0.748	0.201

Mitochondrial Beta-Oxidation of Medium Chain Saturated Fatty Acids	0.398	0.400	1	0.748	0.650
Retinol Metabolism	0.415	0.382	1	0.750	0.082
Ethanol Degradation	0.438	0.358	1	0.750	0.515
De Novo Triacylglycerol Biosynthesis	0.447	0.350	1	0.750	0.200
Ketone Body Metabolism	0.447	0.350	1	0.750	0.705
Glycerolipid Metabolism	0.459	0.338	1	0.750	0.437
Inositol Phosphate Metabolism	0.462	0.335	1	0.750	0.372
Inositol Metabolism	0.462	0.335	1	0.750	0.255
Butyrate Metabolism	0.463	0.334	1	0.750	0.719
Cardiolipin Biosynthesis	0.492	0.308	1	0.784	0.299
Phospholipid Biosynthesis	0.531	0.275	1	0.812	0.272
Pantothenate and CoA Biosynthesis	0.534	0.272	1	0.812	0.069
Betaine Metabolism	0.576	0.240	1	0.859	0.492
Amino Sugar Metabolism	0.627	0.203	1	0.921	0.404
Porphyrin Metabolism	0.679	0.168	1	0.941	0.044
Homocysteine Degradation	0.693	0.160	1	0.941	0.500
Spermidine and Spermine Biosynthesis	0.695	0.158	1	0.941	0.429
Threonine and 2-Oxobutanoate Degradation	0.698	0.156	1	0.941	0.000
Methionine Metabolism	0.719	0.143	1	0.941	0.240
Histidine Metabolism	0.736	0.133	1	0.941	0.759
Lactose Synthesis	0.767	0.115	1	0.941	0.728
Glycolysis	0.768	0.115	1	0.941	0.442
Starch and Sucrose Metabolism	0.781	0.108	1	0.941	0.235
Nucleotide Sugars Metabolism	0.782	0.107	1	0.941	0.931
Glycerol Phosphate Shuttle	0.787	0.104	1	0.941	1.000
Taurine and Hypotaurine Metabolism	0.797	0.098	1	0.941	0.000
Pyrimidine Metabolism	0.799	0.098	1	0.941	0.634
Androstenedione Metabolism	0.803	0.095	1	0.941	0.178
Pterine Biosynthesis	0.808	0.093	1	0.941	0.156
Ubiquinone Biosynthesis	0.820	0.086	1	0.941	0.000
Androgen and Estrogen Metabolism	0.822	0.085	1	0.941	0.186
Estrone Metabolism	0.825	0.083	1	0.941	0.160
Fructose and Mannose Degradation	0.831	0.080	1	0.941	0.221
Pentose Phosphate Pathway	0.849	0.071	1	0.950	0.640
Steroidogenesis	0.884	0.053	1	0.954	0.107
Sulfate/Sulfite Metabolism	0.917	0.037	1	0.954	0.000
Trehalose Degradation	0.926	0.033	1	0.954	0.211
Lactose Degradation	0.926	0.033	1	0.954	0.000
Riboflavin Metabolism	0.926	0.033	1	0.954	0.000
Thiamine Metabolism	0.926	0.033	1	0.954	0.000
Sphingolipid Metabolism	0.936	0.029	1	0.954	0.114
Biotin Metabolism	0.943	0.025	1	0.954	0.000
Vitamin K Metabolism	0.944	0.025	1	0.954	0.000
Folate Metabolism	0.956	0.020	1	0.956	0.104
